# Toward the Allelopathy of *Peganum* sp. and Related Chemical Constituents in Agriculture

**DOI:** 10.3389/fpls.2021.796103

**Published:** 2022-01-21

**Authors:** Gabin Thierry M. Bitchagno, Mustapha El Bouhssini, Ismail Mahdi, Jane L. Ward, Mansour Sobeh

**Affiliations:** ^1^AgroBiosciences Research Division, Mohamed IV Polytechnic University, Ben Guerir, Morocco; ^2^Department of Computational and Analytical Sciences, Rothamsted Research, Harpenden, United Kingdom

**Keywords:** secondary metabolites, weed management, pest control, allelochemicals, bioprotection, Zygophyllaceae (Nitrariaceae)

## Abstract

The genus *Peganum* constitutes one of the perennial groups of plants of semi-arid regions across the world. It produces diverse classes of metabolites with claimed valuable pharmacological applications. Despite the key chemical and biological properties of the genus, its allelopathy or that of one of its species has not been reviewed yet. Thus, the present survey aims to report the agricultural applications of extracts, fractions, and compounds from the genus *Peganum*. This work was based on the available literature related to both the *Peganum* genus and agriculture, which were generated from available high-impact scientific engines. The plants in this genus contain a large group of secondary metabolites including phenolic compounds, terpenes, and *N*-containing compounds. Alkaloids, as the main components of the extracts from plants in the genus, were identified as the major active principles. The toxicity of *Peganum* isolates against plants and related pest organisms was also reviewed. Extract preparations from species of *Peganum* were listed among insecticidal and herbicidal allelochemicals used for crop protection. The review also tried to contextualize natural products in agriculture. *Peganum* plant extracts and fractions have showed significant potential in weed and crops management, soil health, and biopesticide production.

## State of the Art

Natural products (NPs) are molecules produced by living organisms found in nature (Cutler and Cutler, 2000; Bitchagno et al., 2020). Their interests in medicine are of common knowledge. Natural organisms like plants and microbes produce compounds that are not important for their primary metabolism but can be exploited in defense against various attacks including insects and herbivores (Bitchagno et al., 2015; Mbaveng et al., 2019; Nganou et al., 2019a). This self-protection predisposition in nature can be used to inform the development of new plant protecting agents. In 1996, the International Allelopathy Society defined allelopathy as a process involving bioactive secondary metabolites from various organisms (e.g., plants, microorganisms, viruses, and fungi) that influence the growth and development of other organisms in agriculture and biological systems. These biomolecules are known as allelochemicals, and they have beneficial or detrimental effects on the target organisms (Anaya, 2006). However, allelochemicals are not nutritional compounds produced by secondary metabolism and belong to different chemical classes, of which the most important are phenols and terpenoids.

In agriculture, allelopathy can be used to improve crops and food production by targeting either weeds or insects, which negatively influence the growth and development of plants. Historically, synthetic molecules have been the first choice of chemicals for pest and plant pathogen control (Duke and Lydon, 1987). However, the use of synthetic insecticides has given rise to many ecological concerns, including toxic residues in the environment, which are harmful to mammals and other organisms (Duke and Lydon, 1987). Some of these chemicals affect our central nervous system, inducing non-degenerative diseases and related illnesses. They can also affect other aspects of the human central and nervous system (Duke and Lydon, 1987).

One of the sustainable development goals (SDGs) launched by the United Nations (UN) was a recommendation to produce and eat safe foods to foster development (SDG 2 and SDG 12). That is, rather than applying synthetic chemicals to farm fields, the SDGs encourage the use of biodegradable and bio-related materials to control the germination, seedling development, and all other related stages of plant growth and food storage and, consequently, to improve how we feed ourselves and others. In this vein, allelopathy principles and applications in agriculture become obvious to develop further. Since ancient times, plants and materials thereof have been used in agriculture to combat insects and weeds and in fact people did not wait for the UN recommendations to start using materials readily available in their immediate environment to control crop and food production (Duke and Lydon, 1987). The genus *Peganum*, for instance, is known for its large spectrum of bio-related activities including its applications in pest control in countries of the Sahara regions. The present work aims to sum up reported data in the literature on the uses of *Peganum* sp. to promote plant growth and food storage management issues. It also intends to critically address the opportunity of applying NPs in agriculture. The context of the research in Africa in respect to the subject is discussed. This survey follows our continued search for the application of plant extracts and constituents as principal feedstocks in the development of drugs and allelochemicals (Koagne et al., 2017; Nganou et al., 2019b; Tchinda et al., 2019; Mbaveng et al., 2020; Ben Mrid et al., 2021; Damen et al., 2021). The core of pool documents examined for this review was provided from SciFinder-n and PubMed or ScienceDirect when entering the references “*Peganum* and activities” and refining the search with different concepts including fungicides, proteins, growth and development, plant, insecticides, lipid peroxidation, herbicides, phytopathogens, seedling, and phytotoxicity. A total of 89 research items were then generated, of which 70 reported one of the listed concepts.

## The Genus *Peganum*

The genus *Peganum* is a group of only five species belonging to the family Zygophyllaceae. However, new developments in the phylogeny of the genus suggests moving the *Peganum*, *Nitraria*, and *Tetradiclis* genera from their initial collocation to a new one termed Nitrariaceae (Zhang and Chi, 2019). There is still discussion on this improvement, and one can notice that even though the recommendation was first proposed in 1996, the scientific community is still defining the genus *Peganum* with its initial botanical characteristics (Sheahan and Chase, 1996). The origin and geographical distribution of *Peganum* species are quite diverse.

The most popular species in the group, *Peganum harmala*, originates from the Mediterranean region, Southeastern Europe. It is also believed to have been mentioned in the Persian ancient cultures as Avestan *haoma* (Monsef et al., 2004; Mekki, 2014). It has been claimed to have habitats in Nord Africa Sahara regions, in Eastern countries including Iran, Iraq, Turkey, China, and Pakistan, and in the Mediterranean regions across Europe (Spain, Italy) (Monsef et al., 2004; Mekki, 2014). Other species of the genus include *Peganum mexicanum* originating from Mexico, *Peganum nigellastrum* and *Peganum multisectum* whose first specimens were discovered in Mongola, China, and *Peganum taxanum* endemic to Southern North America. Only few notes are available for other species of the genus, especially *P. harmala* (Zhao et al., 2011).

*Peganum harmala* is an all-purpose plant whose application in folk medicine of Eastern Mediterranean regions is broad and diverse. It exists in the literature under various trivial names, including African rue, Syrian rue, wild rue, esfand or espand, or harmel. It was mainly used as an aphrodisiac and exploited during traditional rituals (Apostolico et al., 2016), although many other applications in folk medicine are known. For instance, dried capsules of the plant are often hung in homes or even in vehicles to protect from evil eye in Turkey while the Moroccans use it against *Djinn*. Indians applied the roots to eliminate body lice, and the powder from seeds is exploited in Greece as an antiparasitic agent against tapeworms and to alleviate fever. Various plant extracts have been reported for their antimicrobial, antifungal, analgesic, and antitumor activities (Apostolico et al., 2016). The reference *Peganum* in SciFinder-n gives rise to more than 1,300 results, among which there are 42 reviews and one book, while in ScienceDirect, a search returns 147 reviews and more than 167 book chapters. However, to the best of our knowledge, this study is the first review reporting the application of different organs of plants from the genus *Peganum* to control the development of crops and for food management.

## Chemical Distribution in *Peganum* Genus

**FIGURE 1:**
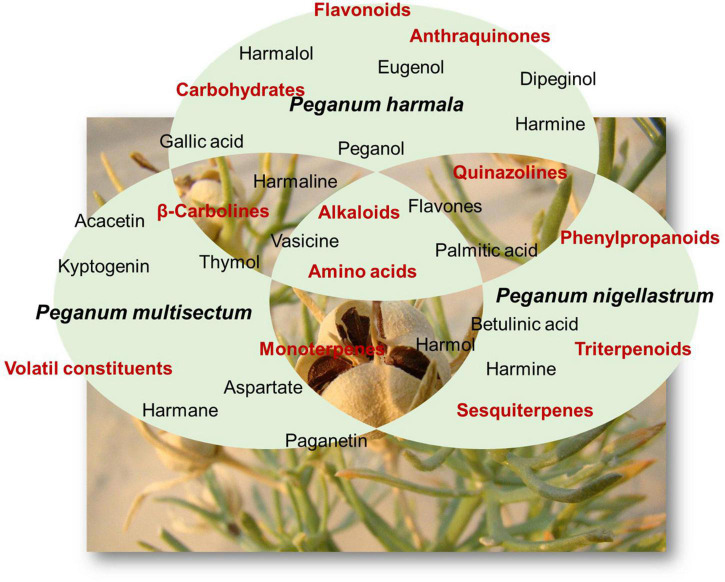
Main chemicals and families of compounds found in *Peganum* sp.

Most studies in the literature concern only *P. harmala*. Li et al. (2017) have drawn up an up-to-date list of chemical constituents of the genus *Peganum*, at least until 2017. Several compounds listed occurred in species other than *P. harmala*. The main compounds occurring in *Peganum* are alkaloids, flavonoids, phenylpropanoids, triterpenoids, anthraquinones, carbohydrates, amino acids, and volatile constituents (Figure 1; Li et al., 2017). Phytochemical screening of the leaves of *P. harmala* showed the presence of saponins, steroids, and tannins (Pahlavia et al., 2018). The alkaloids are sorted into two types, namely, β-carbolines and quinazolines (Li et al., 2017). *P. harmala* alkaloids are more heavily distributed in seeds compared with other organs and are found mostly in ripe rather than in unripe seeds (Kamel et al., 1970; Li, 1996; Kartal et al., 2003; Abbasipour et al., 2010).

β-Carboline-type alkaloids are the most reputed in the genus and claim to be the chemical marker of the genus. Their members are among the most abundant in terms of their mass in each plant, including harmane, harmol, harmine, and harmaline (Li et al., 2017). β-Carbolines occurred or have been reported only in seed organs, while the most reported constituents from the aerial parts are quinazolines (Li et al., 2017). However, both types of alkaloids are distributed in aerial parts of other species, including *P. multisectum* and *P. nigellastrum*. In addition, some alkaloids have not been reported in *P. harmala* yet but were found only in *P. multisectum* (quinalizine and 9-amino-2,3,5,6,7,8-hexahydro-1*H*-cyclopenta [b]quinoline) and *P. nigellastrum* (nigellastrine I and nigellastrine II) (Li et al., 2017).

Flavonoids are distributed in *Peganum* species in both seeds and aerial tissues. They are almost all present in *P. harmala*, except 7,4′-dihydroxy-3′-methoxy-5-*O*-rutinoside occurring in *P. multisectum* and diosmetin 7-*O*-β-D-glucopyranosyl(1→2)-β-D-glucopyranosyl(1→2)-[α-L-rhamnopyranosyl(1→6)]-β-D-glucopyranoside in *P. nigellastrum* (Li et al., 2017). Triterpenoids and phenylpropanoids, in contrast, are exclusively present in the roots of *P. nigellastrum* for the former and the aerial tissues for the latter (Li et al., 2017). The pentacyclic triterpene 3α-acetoxy-27-hydroxyolean-12-en-28-oic acid methyl ester has been found in the seeds of *P. harmala* (Li et al., 2017). Anthraquinones have only been reported in the seeds of *P. harmala*, whereas carbohydrates are claimed to be present in both aerial parts and seeds of *P. harmala*, exclusively (Li et al., 2017). The species *P. harmala*, *P. multisectum*, and *P. nigellastrum* contained 17 out of 20 essential amino acids. Only glutamine, asparaginine, and tryptophan are absent (Li et al., 2017). This helps to understand the reason why many alkaloids occur in the genus.

Phenolic acids have been detected in the aqueous extracts of leaves of *P. harmala* and include gallic, vanillic, caffeic, syringic, and *trans*-ferulic acids as well as benzoic acid derivatives (Sodaeizadeh et al., 2009). Only four of them were found in the stems with caffeic acid predominating while the roots only contained gallic acid, 4-hydroxybenzoic acid, syringic acid, and cinnamic acid. 4-Dihydroxybenzoic acid was the highest component in the leaves and roots of the plant extract (Sodaeizadeh et al., 2009).

Gas chromatography-mass spectrometry (GC-MS) analysis of a seed extract of *P. harmala* revealed the occurrence of 2-undecylcyclopropanepentanoic acid methyl ester, *trans* 5-octadecenoic acid methyl ester, linoleic acid ethyl ester, leptaflorine, and harmine (Aihetasham et al., 2015; Moussa and Almaghrabi, 2016). Moreover, Apostolico et al. (2016) have examined the essential oils of *P. harmala* and have concluded that oxygenated monoterpenes and sesquiterpenes were paired with non-terpenoid compounds. The latter constitutes the main composition of an oil from the species. The composition of the oils relies, however, on the ecosystem where the plant has grown, and Apostolico et al. (2016) proved this by comparing the composition of five essential oils of the same plant, *P. harmala*, harvested from five different regions, namely, Morocco, Algeria, Egypt, Libya, and Tunisia. The oils contained eugenol as main ingredient (13–70%), followed by thymol, which in certain cases, such as in Morocco, was the major compound and eugenol was second most abundant (Tahrouch et al., 1998). The Algerian species of *P. harmala* was the richest in eugenol, followed by the Libyan and Moroccan samples (Apostolico et al., 2016). Tocopherol derivatives also occurred in the seeds extract of *P. harmala* (Hajji et al., 2020). Moreover, δ-tocopherol (90%) was found to be the most abundant in the series, followed by γ-tocopherol and α-tocopherol. In the series of fatty acids, linoleic acid (66%) was the most abundant, followed by oleic, palmitic, and stearic acids successively (Hassani and El Hadek, 1999; Hajji et al., 2020). The application of these compounds and others in agriculture is dedicated to either protect the crops from pests and weeds attacks or stimulate their growth in their environment.

## Bioprotection Applications of *Peganum* sp. in Agriculture

**FIGURE 2:**
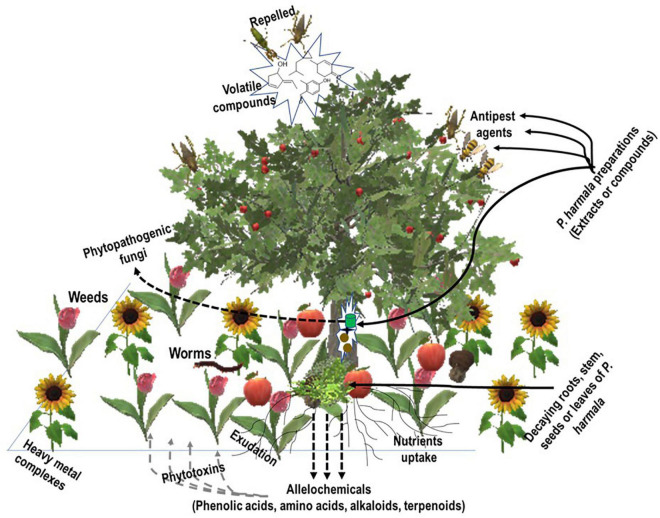
Crops in the rhizosphere. Crops expel pests by releasing volatile and aromatic compounds while exuding (alongside weeds) some chemicals into the soil. Amended soil with mulches of the allelopathy plant enriches it with alkaloids, phenolic compounds, and terpenes, which also constitute phytotoxins for weeds. Preparations of the allelopathy plant (*P. harmala*), extracts, or compounds constitute anti-pest and antipathogenic fungi agents to protect the crop.

NPs constitute an important source of substances for the fight against pests, weeds, and plant fungal threats. In this regard, studies have been reported in the literature about the use of *Peganum* sp. and some of its constituents as allelochemicals with insecticidal, larvicidal, repellent, herbicidal, and antiphytopathogenic fungal properties (Table 1 and Figure 2).

**TABLE 1:** Effects of *Peganum harmala* toward insects and worms.

Contact/oral toxicity	Pests	Larval stages/ Adults	Extract/ compounds	Lethal dose	Mortality rate repellent index	Lethal time/ lethal concentration	Effect on the development stages	References
Oral toxicity	*Tribolium castaneum*	5th instar Adults	Powder fruits	30% in diet		LT_50_ = 6.8 days LT_50_ = 12.6 days		Bounechada and Arab, 2011
		
Oral toxicity		22-days old	MeOH extract (seeds)	50% in diet	58%		Total suppression of F1 adults progeny Lower down weight rate by 50% in 8 days Increase larval pupation period Reduce emergence rate of adults	Jbilou et al., 2008

Contact toxicity	*Tribolium castaneum* *Rhyzopertha dominica*		MeOH extract (Seeds) Alkaloid mixtures	3.5 mg/Kg		34 μg/cm^2^ 24 μg/cm^2^	Lower F1 prigeny population Life span of 70–82 days	Nenaah, 2011

Oral toxicity	*Tribolium castaneum* *Aphis fabae*, *A. gossypii* and *A. nerii*, *Myzus persicae*		Acetone extract (seeds)	60–120 μg/mL	71–95%			Salari et al., 2012

	*Myzus persicae*	Adults			53–73%			

Oral toxicity	*Schistocerca gregaria*	5th instar	EtOH extract (seeds)				Egg-laying delay of 8 days, a decrease in hatching rate, a 70–100% mortality after 5–16 days from the first exposure and a severe impact on the developmental stages of the females including loss of weight and water	Abbassi et al., 2003 Kemassi et al., 2012 Idrissi Hassani and Herms, 2008
		
Oral toxicity		5th instar Adults	Essential oil (leaves)			LT_50_ = 6 min LT_90_ = 19 min		

Oral toxicity	*Locusta migratoria*		MeOH extract (areal)	2% in diet			Reduce the fecundity period by 27% Reduce the fertility percentage to 10% Delay the time for first oviposition in adults by 6 days later normal life population Decrease the number of eggs per ootheca 27/43 for control	Abdellaoui et al., 2014
		
Oral/contact toxicity			H_2_O extract (seeds)	30–240 μg/mL	60%		Deformation of wings, 6 days delayed in larval molt, fledging block, pigmentation and increase preoviposition, only two lays for females and a small number of eggs produced	Benzara et al., 2013

Contact toxicity	*Bemisia tabaci*	Larvae to adults	10 min decoction		50% with larvae and no effect with adults		No repellent activity with adults after 3 h of treatment	Al-mazra’awi and Ateyyat, 2009

	*Eretmocerus mundus*				12–15%			

	*Spodoptera littoralis*	3rd stage	EtOH extract, petroleum ether, EtOAc and chloroform fractions		13–100%			Shonouda et al., 2008

	*Microplitis rufiventris*		EtOAc and chloroform fractions		> 70%			

	*Frankliniella occidentalis*		EtOH extract (seeds)					Razavi and Ahmadi, 2016

Oral toxicity	*Heterotermes indicola*		EtOH extract (seeds)		10%	LT_50_ = 3.19 days		Aihetasham et al., 2015

Contact/oral toxicity	*Trogoderma granarium*	3rd instar Adults	Essential oil (seeds)	23.5 μg/mL 50 μg/mL	66–58%		No emergence of adults regardless the type of exposure	Zeinab and Abdelhafiz, 2019

Contact toxicity	*Holotrichia serrata*		2 weeks fermented aerial	1–5 μg/mL	22%/year			Ayub et al., 2021

	*Helicoverpa armigera*		H_2_O, EtOH, benzene extracts (seeds)		47–80%			Dhumad et al., 2015

Oral toxicity	*Drosophila melanogaster*		Leaves decoction	300 μg/mL	90%		Decrease the number of laid eggs and mating	Elbah et al., 2016

	*Aedes aegypti*	4th instar	Essential oil (aerial)			LC_50_ = 101 μg/mL LC_95_ = 146 μg/mL		Yang et al., 2020

	*Plutella xylostella*		EtOH extract (seeds)	30–40 mg/mL			Provoke dead, larval and pupal weigh losses, high percentage oviposition deterrence and lowering egg hatching percentage	Abbasipour et al., 2010

	*Ectomyelois ceratoniae*	4th instar Adults		25–100%	57%	LT_50_ = 2.6 days for larvae LT_50_ = 1.45 days for adults		Nia et al., 2019 Ismahane et al., 2016

### Insecticidal, Larvicidal, and Repellent Properties

*Peganum* species are mostly used in agriculture for their insecticidal, larvicidal, and repellent properties. However, *P. harmala* is almost the only species of the genus to show activity against pests and to exterminate the corresponding larvae. Overall, various pests, including *Sopdoptera littorali*, *Sopdoptera exigua*, *Schistocerca gregaria*, *Rhyzopertha dominica*, and *Tribolium castaneum*, have been screened. Crude organic extracts of *P. harmala* and plant essential oils have proved to be active.

Interests in securing stored grains and foods are as sensible as the time and logistics relevant to ensure seedling growth and germination in the field. *T. castaneum* (Herbst) commonly called red flour beetle is one of the world-known pests of stored foods, especially stored grains. In contrast, the lesser grain borer, *R. dominica*, is recognized among the pests in stored foods. *R. dominica* essentially affect store bought products and stored cereal grains worldwide. It is also a major pest of peanuts (Edde, 2012).

The powder from the fruits of *P. harmala* showed lethal times (LT_50_) at a concentration of 30% (with diet) after 6.8 days for 5th instar larvae and 12.6 days for adults of stored grains pests *T. castaneum* (Bounechada and Arab, 2011). Jbilou et al. (2008) have also assessed the oral toxicity effects of a methanol extract of *P. harmala* seeds on 22-day-old larvae of *T. castaneum* starved for 24 h prior to the experiments. *P. harmala* slowed down the weight rate of larvae fed with diet by 50% in 8 days (Jbilou et al., 2008). It also induced severe effects with 58% of mortality in larvae and a significant effect on the progeny production by totally suppressing the number of F1 adults that emerged from treated medium (Jbilou et al., 2008). It increased, however, the larval period prior to pupation and was not effective in reducing the emergence of adults, with results comparable to the control. The extract was also more potent in the inhibition of the action of α-amylase with halos of 0.9/4.8 mmol/min/larva compared with the control-only made of diet (Jbilou et al., 2008). Similar results have been gathered by Nenaah (2011) in evaluating the toxicity, growth inhibitory, and effects on the progeny production of *P. harmala* and related seed alkaloids against *T. castaneum* and *R. dominica* over both contact and oral toxicities.

The toxicity of the methanol crude and alkaloidal extracts has been shown to be comparable toward both insects with roughly an LC_50_ of 24 μg/cm^2^ against *R. dominica* and 34 μg/cm^2^ on *T. castaneum* (Nenaah, 2011). The activity was more pronounced orally compared with contact toxicity, and extracts should be mixed with diet for a better action. The insecticidal potentials of the extract were time-dependent and increased with the time of exposure (Nenaah, 2011). The F1 progeny production of both stored-grain insects was highly affected with 3.5 mg/kg doses of *P. harmala* extract. The developmental stages of the larvae were also highly affected (Nenaah, 2011). The life span of *T. castaneum* was 81.3, 74.6, and 70.0 days with an alkaloid extract, harmaline and harmine mixture, and a harmaline and harmane combination, respectively (Nenaah, 2011). Likewise, roughly half of the population of the F1 progeny adults compared with approximately 50–80% of the 2nd instar larvae of *R. dominica* were lost when treated with the same dose of either a crude alkaloid extract, a harmaline and harmine mixture, a methanol extract, or a mixture of harmaline and harmane (Nenaah, 2011).

In addition to *T. castaneum*, Salari et al. (2012) have evaluated the toxic and repellent activities of an acetone extract of the seeds of *P. harmala* on various species of *Aphis* genus, namely, *Aphis fabae*, *Aphis gossypii*, and *Aphis nerii* and on *Myzus persicae*. Together with other *Aphis* species, *M. persicae* is one of the most important vectors in the transmission of plant virus diseases. The repellent bioassay was conducted only on *M. persicae*. All bioassays were conducted at 60 mg/ml for Aphids and *M. persicae* and at both 60 and 120 mg/ml for *T. castaneum* (Salari et al., 2012). The mortality rate in the Aphids community was more pronounced on *A. gossypii* (95%) than the others (71% on *A. fabae* and 80% on *A. nerii*) after 72 h of the first exposure (Salari et al., 2012). Meanwhile, up to 90% of *M. persicae* also died after the same delay and at the same concentration (Salari et al., 2012). The effect of the acetone extract on the red flour pests was not time-dependent since the susceptibilities of the insect were almost stable at 60 mg/ml (8%) and 120 mg/ml (27%) for any of the delays applied in the study. Additionally, the repellent index of the extract against *M. persicae* increased to 73% for early birth insects and to 53% for at least 3-day-old adults (Salari et al., 2012).

Another most important pest whose susceptibility has been studied against *Peganum* species is the so-called desert locust, *S. gregaria*. It is one of the most distributed pests across continents and thus a serious threat for agricultural production. The ethanol extract of seeds of *P. harmala* was applied in feeding the desert locusts, looking at its effect on the 5th instar larvae and on ovarian growth of insects (Abbassi et al., 2003). The extract expressed an egg-laying delay of 8 days, a decrease in hatching rate, a 70–100% mortality after 5–16 days from the first exposure, and a severe impact on the developmental stages of the females including losses of weight and water (Abbassi et al., 2003). The leaf essential oil of *P. harmala* has been evaluated for its toxicity on the desert locust (Abbassi et al., 2003). The 5th instar larvae and adult insects showed similar behavior when treated with the crude oil and with common insecticides. The lethal times LT_50_ on larvae and adults (Abbassi et al., 2003) were 6 and 19 min, respectively. Similar results have been recorded by Kemassi et al. (2014) when applying the leaf essential oil of *P. harmala* on the larvae of desert locust at the same stages of their development, with lethal times at 50% of 6 min and at 90% of 19 min (Kemassi et al., 2014). The toxicity of extracts of *P. harmala* against the *S. gregaria* was attributed to necrosis of intestinal and related tissues (Idrissi Hassani and Herms, 2008).

Studies have also been conducted on the migratory locust, *Locusta migratoria*, one of the species of the *Locust* genus and recognized as the most widespread species in the group (Oonincx et al., 2010). The methanol extract of the aerial part of *P. harmala* influenced, at a concentration of 2% (in diet), the reproduction events in females of *L. migratoria*, reducing the fecundity period by 27% and the fertility percentage to 10% (Abdellaoui et al., 2014). The time for first oviposition in female adults was delayed to 18, 6 days later than normal life population, whereas the number of eggs per ootheca decreased to 27 compared with 43 for the control (Abdellaoui et al., 2014). The extract at 1% in the diet showed a similar effect to results at 2% (Abdellaoui et al., 2014). In contrast, the aqueous extract of *P. harmala* seeds at various concentrations (from 30 to 240 μg/ml) were applied on *L. migratoria* (Benzara et al., 2013). The mortality dose of 240 μg/ml of extract was at 60% after 3 days from either contact or ingestion treatment (Benzara et al., 2013). As observed on other pests, ingestion treatment has been recorded as more harmful, inducing physiological changes like deformation of wings, 6 days delay in larval molt, fledging block, pigmentation and increased preoviposition, only two lays for females, and a small number of eggs produced (50 compared with 63 eggs for control) (Benzara et al., 2013).

Al-mazra’awi and Ateyyat (2009) examined the efficacy of 10 min decoction of *P. harmala* in water toward different growing stages of the insect *Bemisia tabaci*. The plant extract provoked roughly 50% mortalities of the immature pests but was not active against the adult stage. The difference in activity has been related to the experimental protocol since the immature whiteflies were immersed in the plant extract while the adults were not (Al-mazra’awi and Ateyyat, 2009). As a result, the *P. harmala* extract exerted its insecticidal activity by contact with the pests rather than orally. Accordingly, the extract of *P. harmala* was not able to repel adult whiteflies on tomato leaves after 3 h of treatment (Al-mazra’awi and Ateyyat, 2009). However, the extract was less active against the parasitoid of *B. tabaci*, *Eretmocerus mundus* inducing death only 12–15% in adult parasitoid colonies (Al-mazra’awi and Ateyyat, 2009). This selectivity of *P. harmala* on adult specimens (compared with immature) could be related to the preparation of the active extract. Repellent potential is known to occur due to aromatic or volatile terpenes identified as hydrophobic substances and, therefore, could not be found in the decoction extract of the plant. Likewise, the ethanol extract and related petroleum ether (PE), EtOAc, chloroform-soluble fractions have been evaluated for their toxicity against both the cotton leaf worm, *S. littoralis*, and its parasitoid *Microplitis rufiventris*. Assays were undertaken on the 3rd stage larvae feed during 1–2 days by either the crude extract or each of the fractions (Shonouda et al., 2008). The mortality rates of larval were 33–54%, 33–74%, 40–100%, and 13–47% in 2 days for concentrations ranging from 5 to 20% of crude extract, EtOAc, chloroform, and PE, respectively, the highest values being recorded at the highest concentration (Shonouda et al., 2008). Accordingly, the adult emergence percentages were relatively high for worm treated with crude extract (60–47%) and PE (87–54%), while it was low for treated insects with EtOAc (67–20%) and chloroform (60–00%) (Shonouda et al., 2008). The lowest active concentration of EtOAc and chloroform fractions against the third larval instars was used to evaluate the number of emerged parasitoids 2 days after the first exposure. The chloroform fraction was slightly more active than the EtOAc fraction, with 23 and 25% emerged parasitoids, respectively (Shonouda et al., 2008). Nonetheless, the ethanol extract from *P. harmala* seeds was evaluated for its control capability of flower bugs *Frankliniella occidentalis*. The extract was applied in combination with *Orius horvathi*, one of the natural enemies of bug (Razavi and Ahmadi, 2016). *P. harmala* showed similar activity as the natural enemy. However, the extract was harmful to the natural enemy of bug, making the extract not suitable for an integrated pest management (IPM) of flower thrips (Razavi and Ahmadi, 2016). The ethanol extract of seeds also showed an insecticidal effect on the termites *Heterotermes indicola* with a LT_50_ performance of 3.19 days at a concentration of 10% of extract (Aihetasham et al., 2015). Both contact and ingestion toxicities of the essential oil from seeds of *P. harmala* were recorded on adult individuals of *Trogoderma granarium* (Khapra beetle) (Zeinab and Abdelhafiz, 2019). When fed with grains treated by *P. harmala*, the third instar larvae expressed up to 66% mortality in 3 days at 40 μg/ml with a calculated lethal dose (LD_50_) of 23.5 μg/ml (Zeinab and Abdelhafiz, 2019). Likewise, the oil induced a contact mortality rate of 58% at the same concentration with a LD_50_ of 50 μg/ml. No adults emerged at this concentration for both types of toxicity (Zeinab and Abdelhafiz, 2019). Two-week-old fermented material of the aerial part of *P. harmala* was applied to evaluate the infestation rate of potato by white grub larvae during the seasons 2018 and 2019 (Ayub et al., 2021). The tested material of *P. harmala* induced approximately 22% of infestation of potato tubers annually at diluted solutions of 1–5 ml/L similar to the yield loss of a chemical insecticide used as control (Ayub et al., 2021). The diluted (20–30%) aqueous, ethanol, and benzene extracts of *P. harmala* seeds were applied against *Helicoverpa armigera*, a tomato fruit borer insect (Dhumad et al., 2015). The benzene extract was the most active extract after a day of first exposure, inducing up to 80% of mortality followed by the ethanol extract (67%) and the aqueous extract (47%) (Dhumad et al., 2015). Decoction of leaves of *P. harmala* induced negative behavioral sequences in mating adults of fruit fly (*Drosophila melanogaster*), provoking up to 90% of abortion at a concentration of 300 μg/ml (Elbah et al., 2016).

A repellent activity was encountered against the insects, causing a decrease in the number of laid eggs, thus reducing mating (Elbah et al., 2016). Limonene (15%) and thymol (12%) were abundant in the essential oil from the aerial part of *P. harmala* (Yang et al., 2020). The LC_50_ and LC_95_ indices of the essential oils against the fourth instar larvae of *Aedes aegypti* were evaluated to be 101 and 146 μg/ml, respectively, whereas thymol was the most potent, meaning that there should be antagonistic effects in the activity of *P. harmala* (Yang et al., 2020). The EtOH extract of *P. harmala* seeds expressed diverse effects toward *Plutella xylostella*. Extracts exhibited a larvicidal activity in a concentration-dependent manner (Abbasipour et al., 2010). Roughly, 30–40 mg/ml of the extract was sufficient to provoke death, larval and pupal weigh losses, high percentage oviposition deterrence, and lowering egg hatching percentage (Abbasipour et al., 2010). The contact toxicity of aqueous extracts of seeds *P. harmala* was assessed on the egg hatching and larvae development of *Ectomyelois ceratoniae* (Nia et al., 2019). Regardless of the concentration (25–100%) of extracts, the toxicity was almost clear on egg hatching and did not exceed 8% in respect to the mortality rate of the 3rd and 4th larvae (Nia et al., 2019). It also provoked 57% mortality in 5 days post-exposure on the fourth instar larvae and adults of the date moth *E. ceratoniae* with a LT_50_ of 2.6 days for larvae and 1.45 days for adults, which also totally died for the same period of exposure (Ismahane et al., 2016).

### Antiphytopathogenic Microbial Activity

One of the rare species in the *Peganum* genus to be investigated, *P. multisectum*, showed potent capabilities to inhibit the germination and growth of soil-borne fungi and insecticidal activity against eight pathogenic fungi including *Usarium graminearum* Schw, *Sphaerotheca leucotricha* Solm, *Phytophthora capsici* Leonian, and *Puccinia glumarum* Erikss as well as against two Aphids (*Schizaphis graminum* and *M. persicae*) (Jianxin et al., 2006). Three basic plant extracts, i.e., the dissoluble, fat-soluble, and total alkaloids, each at 0.5 mg/ml, have showed activity against the tested pathogens (Jianxin et al., 2006). Roughly, 78% of the fungi were sensitive toward the extracts after 72 h, while the extracts have showed almost the same degree of insecticide, with approximately 40% potential on *S. graminum* and 29% on *M. persicae* after 48 h (Jianxin et al., 2006). Some other works reported on the capability of *P. multisectum* in seedling toxicity or pest control or even against soil fungi (Liu et al., 2004; Jianxin and Guolin, 2005; Xue et al., 2007a,b), but their access was limited. However, mentioning such data in this review is essential since *P. harmala* is almost the only species whose application in agriculture is widespread. One should also be aware of similar activities for *P. multisectum*.

*Ralstonia solanacearum* phylotype II is a pathogen responsible for the brown rot potato found to spread under different climates, including tropical, subtropical, and temperate conditions (Mohamed et al., 2019; Shaheen and Issa, 2020). It is dispersed in other hosts like tomato, pepper, and eggplant. *Pectobacterium carotovorum* also affects potato both in-field and during storage (Perombelon and Kelman, 1980; Czajkowski et al., 2015; Shaheen and Issa, 2020). It causes tuber soft rot and blackleg of potato. *Burkholderia gladioli* causes the yellowing and death in the onion crop (Burkholder, 1950; Yabuuchi et al., 1992; Shaheen and Issa, 2020). *Erwinia amylovora* infects pome fruit trees, damaging blossoms, leaves, fruitlets, shoots, trunks, and limbs (Winslow et al., 1920).

The water-soluble extract of the leaves of *P. harmala* showed considerable capacity to inhibit the growth of 10 isolated phytopathogenic fungi of tomato fruit, including *Alternaria alternata, Alternaria solani, Phytophthora infestans, Fusarium oxysporum* f. sp. *lycopersici, Verticillium albo-atrum, Botrytis cinerea, Colletotrichum coccodes, Rhizopus stolonifer, Rhizoctonia solani*, and *Fusarium solani* (Pahlavia et al., 2018). The susceptibility of the plant extract toward fungi increased with concentration, covering up to 95% of inhibition with a concentration of 200 mg/ml (Pahlavia et al., 2018). Additionally, results highlighted here against 10 pathogenic fungi of the seed oil of *P. harmala* collected in various geographical regions in Tunisia were similar to existing data. Indeed, the seed oil of the plant showed a significant effect on the growth of mycelia of all the fungi with halos ranging from 32 to 83% but no effect was noticed on *Alternaria* sp. (Hajji et al., 2020). The fungi used include *R. solani*, *Macrophomina phaseolina*, *Pythium* sp. 1, *Pythium* sp. 2, *Alternaria* sp., *Colletotrichum* sp., *Monosporascus cannonballus*, *Fusarium solani* f. sp. *cucurbitae*, *Fusarium oxysporum* f. sp. *melonis*, and *Fusarium oxysporum* f. sp. *niveum*.

The total alkaloid extract of *P. harmala* seeds exhibited significant antibacterial activity against the causal pathogen of brown rot in potato with MIC of 4 at 300 μg/ml while the effect was moderately significant on the three other tested bacteria (Shaheen and Issa, 2020). The same concentration of the alkaloid fraction also restored the potato by 68% when treated *in vivo* infected potato. The total alkaloid extract also promoted the growth of tubers and leaves at the same concentration of 300 μg/ml (Shaheen and Issa, 2020).

### Phytotoxicity

A phytotoxic substance also called a phytotoxin is a chemical that is toxic to the plant growth (Günthardt et al., 2018). These chemicals can originate from other plants. Allelochemicals constituted one of the classes of phytotoxins alongside allergens, hallucinogens, fatal toxins, and biopesticides. Their effects on plants are, however, expected to be broad compared with that of a single metabolite. The phytotoxic effect of a plant has also been established to occur during its own decomposition on soil (An et al., 2001). Subsequently, it is submitted to the physicochemical and microbiological properties of the soil (Kobayashi, 2004; Popa et al., 2008). The bioavailability of allelochemicals is related to various parameters, including the ion exchange capacity of soil, pH, organic content, structure, and texture (Scavo et al., 2019). The phytotoxin can, therefore, be either inactivated, overactivated, or converted into other toxins by the soil microorganisms, chemicals, or ions (Kobayashi, 2004). The time of decomposition before seeding is also a valuable parameter to consider recalling that the efficacy of the plant residue decreases with the increasing time of decomposition (Xuan et al., 2005; Sampietro et al., 2007). In addition, crop rotation practices, inter-cropping, and mulching all involve the application of allelochemicals in agroecosystems (Scavo and Mauromicale, 2021).

Various parameters related to the seedling growth of two weed species, namely, *Avena fatua* L. (Poaceae) and *Convolvulus arvensis* L. (Convolvulaceae), were evaluated (Sodaeizadeh et al., 2010) under *P. harmala* material in soil. The extract showed a concentration-dose dependence in reducing each of the parameters tested, including the seedling length, the seedling dry weight, the leaf area, the total chlorophyll amount, and leaf moisture. Apart from the latter which was not sensitive to the application of *P. harmala* to soil, all other parameters were affected (Sodaeizadeh et al., 2010). The leaf residues of the donor were more active than its stem and root residues. The leaf residues reduced up to 64% of seedling length, affecting the seedling dry weight by 87%, the leaf area by 25–90%, and the total chlorophyll amount by 25–50%. *C. arvensis* was more susceptible than *A. fatua* toward *P. harmala* (Sodaeizadeh et al., 2010). Leaves of *P. harmala* improve the capacity of the soil in nitrogen, phosphorus, potassium, manganese, and copper, while stem and roots are limited to nitrogen (Sodaeizadeh et al., 2010). The efficacy of *P. harmala* residues is significant during the first 3 days when the soil contained high level of phenolic compounds although the effects vanished between 7 and 15 days from exposure (Sodaeizadeh et al., 2010).

Based on previous evidence that phenolic compounds induced significant allopathic properties, Sodaeizadeh et al. (2009) investigated this literature assertion by applying both crude extracts and phenolic fractions from different organs of *P. harmala* to the germination and seedling growth of weed plants *A. fatua* L. (wildoat; Poaceae) and *C. arvensis* L. (field bindweed, Convolvulaceae) (Sodaeizadeh et al., 2009). The reductions occurred at a level of 64–72% on *A. fatua* and 27% on *C. arvensis* when grains were treated with equal amounts of extract (Sodaeizadeh et al., 2009). The shoot dry weights (SDWs) of both weeds were not affected by aqueous extracts of roots and stems. Leaf extracts, however, diminished the SDW of *C. arvensis* by 27% (Sodaeizadeh et al., 2009). Regarding the root dry weight (RDW), all of the extracts reduced the weights by approximately 50%. Leaf extracts were thus the most active sample (Sodaeizadeh et al., 2009). A minimum of 16% of leaf extract concentration was sufficient to induce significant inhibition of chlorophyll A in both weeds, while no extracts were active on the concentration of chlorophyll B reduction in weeds (Sodaeizadeh et al., 2009).

Harmine and harmaline isolated from the seed extract of *P. harmala* showed potent inhibition potential on seedling growth of dicot and monocot plants (Shao et al., 2013). Harmaline inhibited the elongation (root and shoot) of lettuce and amaranth by 30–50% at 5 μg/ml, while harmine was less active. The phytotoxicity of harmaline was comparable to that of the total alkaloid extract toward all the plants (Shao et al., 2013). The aqueous extract of leaves from *P. harmala* was screened for its germination and seedling growth inhibition of wheat and mustard (Aslam et al., 2016). For 5–25% dilution of extract, the germination rate of both the crops was more affected by a more highly concentrated extract (20–25%) (Aslam et al., 2016). The germination rate of mustard was reduced to 80% and that of wheat to approximately 53 at 25% after 7 days of the first exposure. The inhibition was, therefore, dose- and time-dependent (Aslam et al., 2016). Harmaline induced a cell growth, pigment content, and oxygen evolution reduction on the green algae *Chlorella pyrenoidosa* (Deng et al., 2014).

## Tentative Allelopathy Mechanisms of Action of *Peganum*-Related Substances

Several studies have examined the mechanisms of action of *Peganum* species and related chemical constituents toward larvicidal or phytotoxic activities (Table 2). The difference in chemical composition of the plant affects the toxicity of plant essential oils toward weed germination and seedling growth. *P. harmala* extracts and isolated alkaloids have been reported to inhibit acetylcholinesterase enzyme (Zheng et al., 2009, 2011; Yang et al., 2015). This property and others among brain-related susceptibilities have been hypothesized as being responsible for many of their activities against pests. For instance, the mechanism of action of the toxicological toxicity of *P. harmala* was assessed *in vivo* through the toxicity of the ethanol extract on the worm, *Caenorhabditis elegans*. Miao et al. (2020) demonstrated the impact of *P. harmala* on the central nervous system and on the insulin/IGF-1 signaling pathway of the worm. *P. harmala* significantly reduced the life span, development, reproduction, and locomotion susceptibilities of the worm after a prolonged exposure to 1 mg/ml of the extract (Miao et al., 2020).

**TABLE 2:** Available mechanisms of action of *Peganum* individuals toward insects and plants.

Individuals	Effects	Proposed mechanism of action	References
β-carboline alkaloids	Toxicological properties	Inactivate receptors of benzodiazepine, imidazoline, serotonin and opiate	Pimpinella and Palmery, 1995; Herraiz and Chaparro, 2005; Miralles et al., 2005; Herraiz et al., 2008
		Inhibition of cytochrome P450 and MAO	
	Scavenging activity	Prevent dopamine-induced mitochondrial damage, and PC12 cell death	Lee et al., 2000
	Antimutagenic and antigenotoxic activity	Inhibit H_2_O_2_, and paraquat	Moura et al., 2007
Harmaline	Larvae growth reduction	Induce glutathione *S*-transferase in pest body fat and midgut tissues	Rizwan-Ul-Haq et al., 2010
		Affect superoxide dismutase and catalase enzyme contents	
	Larvae weight loss	Reduction in protein and glycogen contents and inhibition of α-amylase activity	Rharrabe et al. (2007)
	Insecticidal activity	Cytotoxicity on pest mid-gut epithelia with vacuolization of the cytoplasm	
		Autophagic vesicles and lysosomic structures induction	
		Fragmentation of rough endoplasmic reticulum cisternae	
		Disruption of microvilli and plasma membrane	
		Shedding of the cytoplasmic contents into the mid-gut lumen	
Harmaline and harmol	Acetylcholine esterase (AChE) activity	Inhibit AChE in pests	Zheng et al., 2009
Alkaloids	Phytotoxicity	*P. harmala* (stem and roots) N-containing metabolites induce growth inhibition due to temporary N deficiency in amended soil	Sodaeizadeh et al., 2010
*p*-hydroxybenzoic acid	Phytotoxicity	Inhibit radical growth	Reigosa et al., 1999
Ferulic acid		Chlorophyll reduction	Einhellig and Rasmussen, 1979
Vanillic acid			
Phenolic compounds		*P. harmala* residues (leaves, stem or roots) not affect plant water balance of receiver species, responsible in general for growth inhibition	Sodaeizadeh et al., 2010
Volatile oil		Damage the plant cellular membranes	Shao et al., 2013

The poisonous properties of some alkaloids isolated from *Peganum* species as well as some of their extracts are highlighted in the ancestral medicine of the plant and have been confirmed throughout with scientific investigations (Rizwan-Ul-Haq et al., 2010). Thus, detrimental abilities of *Peganum* materials have been observed in pest control. Indeed, harmaline, one of the main alkaloids of *P. harmala*, induced glutathione *S*-transferase (GST) in body fat and midgut tissues of *S. exigua*; the higher level of GST in tissue is a sign of a pronounced resistance expression of the insects toward xenobiotics (Rizwan-Ul-Haq et al., 2010). GST is an enzyme essential in the detoxification process in pests. Its induction protects internal tissues from damages due to the accumulation and thus the effective action of toxicological drugs (Rizwan-Ul-Haq et al., 2010).

The properties of essential oils could be related to their composition of oxygenated volatile terpenes, including monoterpenes and sesquiterpenes (Shao et al., 2013). Such compounds are claimed to damage the complete formation of plant cellular membranes, with a consequence that cell contents are drained out, inducing a progressive death of the organism (Youmbi et al., 2020; Sonfack et al., 2021). This mechanism can be extended to some herbicidal and antiphytopathogenic activities recorded with *P. harmala* lipophilic extract.

## Another Side of Pesticides and Biopesticides in Agriculture

The therapeutic qualities of NPs and natural preparations are undeniable. In medicine for instance, NPs and related drugs, besides their main purposes, induce the development of many diseases, including cancer, non-degenerative disorders, and other stress-related illnesses. Some of these diseases are induced by a modification of the natural metabolism of a certain process due to the integration of a compound or a gene modification in DNA. Consumable plants are medicines that humans ingest through their foods. As a result, the transformations that could take place in plants due to the use of pesticides or biopesticides may affect human health. Therefore, we should further investigate the possible changes in plant metabolism following treatment with pesticides (Hancianu and Aprotosoaie, 2012).

Pesticides on farms are degraded by chemical, biological, or physical processes, including biotransformation, bioremediation, and mineralization (Hoagland et al., 2000). Biotransformation always occurs through biochemical degradation pathways termed in some cases as co-metabolism. This process tends to render pesticides less harmful and more vulnerable to chemical and biological degradative transformations in the host organisms. Pesticides in soils constitute a source of carbon since microorganisms acquired energy from nutrients found in the soil. They are said to be chemoautotrophs in comparison to photoautotrophs the description attributed to plants that gain energy for their metabolisms from sun light (Hoagland et al., 2000). Some pesticides such as alachlor or glyphosate have been reported to influence the level or the presence of natural secondary metabolites in their living plant organisms (Lydon and Duke, 1989). Glyphosate interferes in the metabolisms of cinnamate inhibiting one of the shikimate synthases, while alachlor brings down the level of flavonoids by binding to certain enzymes in their biosynthetic processes (Lydon and Duke, 1989). Actions of glyphosate on shikimate formation also affect the biosynthesis of phenolic compounds essential in the fight of both plants and humans against reactive oxygen species (ROS). The effect on the level of cinnamate by alachlor and sulfonylureas pesticides also influences the level of terpenoids in plants containing significant quantities of essential oils. Some of the components of these oils intervene in the interspecies or intraspecies interactions between the plant kingdom itself and with microorganisms (Lydon and Duke, 1989).

All these effects and others induce dramatic consequences in human health when treated plants are dedicated to food production. Similar effects are also expected when using biopesticides, even though the degree of harm is far from comparable. Compounds like β-carbolines and quinazolines targeted as the main sources of toxicity of *Peganum* plants could influence the metabolism of certain compounds or simply exist in the plant material since some of them are soluble in the volatile oil component of the plant. Furthermore, hybridization effects are expected to alter chemistry and biology, and these effects may also need to be investigated and their consequences need to be understood.

## Outputs and Outlooks

The present survey has highlighted the utility and allelopathy capacities of *Peganum* tissues and compounds *via* a comprehensive review. Only the bioprotection aspect of the plants has already been reviewed and published, while their biostimulant abilities are still awaited. *P. harmala* and *P. multisectum* are the only species investigated in this line so far, with *P. harmala* more than *P. multisectum*. Each organ of plants has already been studied. The seeds in the *Peganum* group have been established as more potent to protect crops both on farms and post-harvest. The essential oils of the seeds or leaves were also broadly used, and they exhibited repellent potency due to their constitution of volatile and aromatic compounds. These compounds also interfere with the metabolism of certain insects, leading to eventual death. Similar behavior could also be observed against weeds.

The activities of plant preparations differ depending on the solvents utilized. Additionally, different environmental and processing methodologies can also affect the final chemical composition of the plant material and needs to be considered ahead of any potential formulation processes. This discrepancy constitutes a threat to the development of allelochemicals in agriculture (Apostolico et al., 2016). The composition of essential oils is also linked to the age of the plant and mature species are typically poor in volatile constituents. Likewise, a difference in the susceptibility of *P. harmala* has been found to be related to its geographical location. This kind of specificity has already been demonstrated with plant preparations for medicinal purposes. Therefore, by controlling the growth and processing of the plant, one can achieve better quality-controlled material for a better efficacy.

The next generation of research on plants from the *Peganum* genus could then continue to reveal the diversity in the chemistry of the group. Recently, Wang et al. (2018) have highlighted racemates and optical compounds from the seeds of *P. harmala* including dimers of indoles and 2-oxoindoles alkaloids (Wang et al., 2018). These new developments call to mind the complexity and wealth of the chemistry of *Peganum*. Another aspect should focus on the changes in chemical profile as a result of growth × environment × processing conditions. Furthermore, none of the reports regarding the studied genus has revealed the biostimulant potential of one of its plant species. *Peganum* plants are though serious candidates since they are sources of various phenolic compounds.

## Author Contributions

GTMB drafted and reviewed the manuscript. MEB, IM, and JLW revised the manuscript. MS revised the manuscript, designed, and conceived the study. All authors approved the final version.

## Conflict of Interest

The authors declare that the research was conducted in the absence of any commercial or financial relationships that could be construed as a potential conflict of interest.

## Publisher’s Note

All claims expressed in this article are solely those of the authors and do not necessarily represent those of their affiliated organizations, or those of the publisher, the editors and the reviewers. Any product that may be evaluated in this article, or claim that may be made by its manufacturer, is not guaranteed or endorsed by the publisher.
